# Clinical Trial: A Mediterranean Low-FODMAP Diet Alleviates Symptoms of Non-Constipation IBS—Randomized Controlled Study and Volatomics Analysis

**DOI:** 10.3390/nu17091545

**Published:** 2025-04-30

**Authors:** Arezina N. Kasti, Konstantinos Katsas, Dimitrios E. Pavlidis, Emmanouil Stylianakis, Konstantinos I. Petsis, Sophia Lambrinou, Maroulla D. Nikolaki, Ioannis S. Papanikolaou, Erifili Hatziagelaki, Konstantinos Papadimitriou, John Kapolos, Jane G. Muir, Konstantinos Triantafyllou

**Affiliations:** 1 Department of Nutrition and Dietetics, Attikon University General Hospital, 12462 Athens, Greece; kastiare@med.uoa.gr (A.N.K.); katkonstantinos@gmail.com (K.K.); kostas.petsis@hotmail.com (K.I.P.); sophialambrinou@gmail.com (S.L.); maroullanikolaki@gmail.com (M.D.N.); 2 Department of Food Science and Technology, University of Peloponnese, 24100 Kalamata, Greece; d.pavlidis@go.uop.gr (D.E.P.); i.kapolos@uop.gr (J.K.); 3 4th Department of Internal Medicine, Medical School, Attikon University General Hospital, National and Kapodistrian University of Athens, 12462 Athens, Greece; manos_stylia@hotmail.com; 4 Department of Clinical Nutrition and Dietetics, General Hospital of Karpathos “Aghios Ioannis Karpathios”, 85700 Karpathos, Greece; 5 Department of Nutrition and Dietetics Sciences, Hellenic Mediterranean University, 72300 Sitia, Greece; 6 Hepatogastroenterology Unit, 2nd Propaedeutic Department of Internal Medicine, Medical School, Attikon University General Hospital, National and Kapodistrian University of Athens, 12462 Athens, Greece; ispapn@hotmail.com; 7 2nd Department of Internal Propaedeutic Medicine, Medical School, Attikon University General Hospital, National and Kapodistrian University of Athens, 12462 Athens, Greece; erihat@med.uoa.gr; 8 Laboratory of Food Quality Control and Hygiene, Department of Food Science and Human Nutrition, Agricultural University of Athens, 11855 Athens, Greece; kpapadimitriou@aua.gr; 9 Department of Gastroenterology, Monash University, Melbourne 3004, Australia; jane.muir@monash.edu

**Keywords:** irritable bowel syndrome, low-FODMAP diet, Mediterranean diet, quality of life, SCFA, BCFA

## Abstract

**Background:** Approximately 20% of patients with irritable bowel syndrome (IBS) link symptoms to food intake; a low-FODMAP diet is effective in managing these symptoms. **Aims:** To evaluate the effectiveness of the Mediterranean version of the low-FODMAP Diet (MED–LFD) compared to NICE guidelines for IBS and to assess changes in stool volatile compound levels. **Methods:** 108 patients with Rome IV IBS without constipation were randomized into the MED–LFD or NICE group. Primary endpoints included changes in symptom severity and responder rate (reduction of >50 IBS-SSS points) after intervention and at 6 months. Secondary endpoints assessed quality of life, symptom burden, adequate relief, anxiety/depression levels, and adherence. Volatile compound levels were measured using Gas Chromatography/Mass Spectrometry. **Results:** At both time points, the MED–LFD group showed a significantly greater improvement in symptom severity (159 ± 80 vs. 253 ± 94 and 168 ± 117 vs. 245 ± 98), responder rates (84.6% vs. 60.8% and 79.1% vs. 52.3%), and adherence (75% vs. 41% and 45% vs. 7%). Similar results were observed for all secondary endpoints, with no serious adverse events reported. The MED–LFD intervention was the strongest independent predictor of being a responder at the first (OR = 6.66; 95%CI = 1.46, 30.4) and second follow-up (OR = 4.85; 95%CI = 1.31, 17.96). Short and branched-chain fatty acids were significantly reduced at both follow-ups. **Conclusions:** The MED–LFD is superior to NICE recommendations in managing non-constipated IBS symptoms and quality of life. It remains to be proven that reduced volatile compound levels might be an objective marker of response to dietary interventions. ClinicalTrials.gov ID: NCT03997708.

## 1. Introduction

Visceral hypersensitivity, low-grade mucosal inflammation, and neuroinflammation are involved in the pathophysiology of IBS via the “gut–brain” axis [1,2]. In addition, up to 20% of IBS patients associate food with symptoms, such as abdominal pain, nausea, bloating, and diarrhea, suggesting that specific dietary components and gut microbiota may contribute to visceral hypersensitivity development [3]. Foods that can trigger symptoms include: (a) FODMAPs (e.g., fructans, lactose, polyols) that induce osmotic load and bacterial fermentation, leading to bloating, gas, and visceral hypersensitivity; (b) High-fat foods delay gastric emptying and stimulate cholecystokinin, exacerbating nausea and discomfort; (c) Caffeine/alcohol increase gastric acid secretion and disrupt gut-barrier integrity; (d) Spicy foods (capsaicin) activate TRPV1 receptors, triggering visceral pain; (e) Artificial sweeteners (e.g., sorbitol) alter microbiota and induce osmotic diarrhea; and (f) Histamine-rich foods (aged cheeses, fermented products) promote mast cell activation and inflammation [4,5,6,7,8,9,10].

While a balanced microbiota is essential for the normal perception of visceral enteric stimuli [11], evidence also suggests that short-chain fatty acids (SCFAs), such as acetate, propionate, and butyrate, produced during the fermentation of non-absorbed carbohydrates by colonic bacteria with the simultaneous production of colonic gases, such as hydrogen and carbon dioxide, may contribute to the pathogenesis of IBS [12]. When fermentable carbohydrates decrease, SCFA production drops, leading to increased protein fermentation and higher levels of branched-chain fatty acids (BCFAs), such as isovaleric and isobutyric acids, in the colon. Butyrate, the main energy source for colonocytes, provides 70–80% of their energy; when lacking, BCFAs can serve as alternatives. Both butyrate and BCFAs are potent histone deacetylase inhibitors, indicating similar regulatory roles in host cells [13]. Ingested dietary fermentable oligosaccharides, disaccharides, monosaccharides, and polyols (FODMAPs) may increase GI water secretion and enhance fermentation in the colon, producing SCFAs and gases. These effects may induce luminal distension and trigger meal-related symptoms in patients with IBS [1,14]. During the last decade, the exclusion of FODMAPs has become increasingly popular for treating these patients [15]. One network meta-analysis of 13 randomized controlled trials (RCTs) showed that a low-FODMAP diet (LFD) outperforms other dietary treatments to relieve IBS symptoms overall [16]. Concurrently, randomized controlled trials (RCTs) and meta-analyses provide strong evidence for the Mediterranean diet’s (MED) anti-inflammatory properties, attributed to its high levels of phenols, and its ability to improve mental health by alleviating symptoms of depression and anxiety [2,17,18,19].

The idea of combining the LFD and the MED for managing IBS symptoms originated from data indicating that low adherence to the MED has been linked to a higher prevalence of IBS [20,21] and the knowledge that low-grade intestinal inflammation is among the components of IBS pathophysiology [22]. Our study aimed to compare the effectiveness of two dietary patterns: a Mediterranean LFD type (MED–LFD) [23] and a diet based on the National Institute for Health and Care Excellence (NICE) guidelines for managing IBS symptoms and improving quality of life [24]. We also aimed to highlight potential pathophysiological insights of dietary intervention by conducting an exploratory analysis evaluating stool SCFAs/branched-chain fatty acids (BCFAs) level changes in a subgroup of enrolled subjects.

## 2. Materials and Methods

### 2.1. Participants and Study Design

We enrolled outpatients aged 18–65 years with non-constipation IBS from Attikon University General Hospital diagnosed using Rome IV criteria [25], and the Bristol Stool Form Scale (BSFS) [25] with an IBS Symptoms Severity Score (IBS-SSS) >175 [26] during the period from July 2019 to April 2023. We excluded patients with, as follows: red flag symptoms and signs; those requiring specialized diet, probiotic and/or antibiotic treatment 1 month before the study initiation; current hypnotherapy or cognitive behavior therapy; food allergies; an inability to stop antispasmodic and antidiarrheal medications at the initiation of the study; and IBS patients with IBS-SSS ≤175. The failure to meet study obligations also led to exclusion. For participants aged 50 and older, a negative colorectal cancer screening test was required.

Throughout the study, routine medication that could affect IBS symptoms was not permitted. However, participants were allowed to use paracetamol, hyoscine butyl bromide, antispasmodics, and loperamide as rescue medications if their symptoms worsened. We recorded adverse events (AEs) and serious adverse events (SAEs) at every stage of the study. Participants were allowed to withdraw their participation from the study voluntarily at any time.

It was a randomized controlled, parallel-group design study with three time points for evaluation: Baseline (2 to 4 days before the intervention began); first follow-up (4 weeks following the introduction of the dietary plan for the NICE group and at the end of the reintroduction phase for the MED–LFD group, with a two-to-four-day time window); and second follow-up (at 6 months, with a 1-week time window). Evaluations and questionnaires used during the study periods are described in detail elsewhere [24].

### 2.2. Ethics

This randomized controlled clinical trial is registered under ClinicalTrials.gov, Identifier: NCT03997708, and the full study protocol has also been published [27]. The Institutional Review Board of the Attikon University General Hospital approved the study protocol (ΕΒΔ435/19-06-2018). All participants provided written informed consent at the time of enrollment.

### 2.3. Randomization and Masking

We prepared a random allocation sequence using block randomization, with a 1:1 ratio for the control and intervention groups (block size n = 4), by utilizing the “randomizeR” (Randomization for Clinical Trials) package in R software [28]. Participants were informed that both diets might help to reduce symptoms; however, we could not mask the diets. Additionally, the researchers conducting the follow-up evaluations were blinded to which intervention group each patient had been assigned.

### 2.4. Intervention Diets

Patients were randomly assigned to one of two diet groups: the MED–LFD (intervention group) and the NICE diet (active comparator). According to one systematic review and meta-analysis published in 2024 that included countries from the Mediterranean basin (Greece, too), the estimated global FODMAP intake is 19.86 g/d, regardless of health status. It is similar for healthy individuals and those with functional gastrointestinal disorders and other gastrointestinal diseases, with small differences [29]. The LFD involves a dietary intake of less than 3.1 g of FODMAPs per day [30]; our MED–LFD group followed a specific weekly dietary plan with FODMAP consumption ranging from 0.77 to 1.91 g per day, based on the cut-off levels [31] defined by Monash University, enriched with MED elements. The first phase of the study lasted 2 to 6 weeks. Table 1 displays the indicative number of servings for food groups included in the Mediterranean diet pyramid [32] that was incorporated into the MED–LFD diet. In the second phase, patients were gradually reintroduced to foods rich in FODMAPs; their tolerance was tested for 6 to 8 weeks. After completing the reintroduction phase, participants were encouraged to adopt a long-term diet (third phase) that met their nutritional needs, including well-tolerated FODMAPs [33].

Participants in the comparator group received a weekly dietary plan for 4 weeks based on NICE guidelines for patients with IBS [24]; they were monitored for 6 months after the intervention began.

The energy intake for both groups was approximately 1800 kcal. In the MED–LFD group, the nutrient intake consisted of 197 g of carbohydrates, 85 g of protein, and 80 g of fats. Respectively, the NICE group had a nutrient intake of 240 g of carbohydrates, 87 g of protein, and 62 g of fats. Detailed information about the two dietary interventions has been published elsewhere [27].

Dietary instructions for the MED–LFD and NICE diets were provided to all participants by dieticians who had completed extensive training from Translational Nutrition Science, Department of Gastroenterology, School of Translational Medicine, Monash University. This training specifically focused on administering low-FODMAP diets in IBS patients [34]. Participants received detailed weekly meal plans for both dietary interventions [34].

Sample meal plans can be found in Supplementary Table S1. The MED–LFD diet sample scored 11 out of 14 on the Mediterranean Diet Adherence Screener (MEDAS), indicating high adherence to the Mediterranean diet. The NICE diet sample scored 8, indicating moderate adherence [35].

The NICE group had up to three consultations: one at baseline, another 4 weeks after the intervention began, and a third 6 months later. The MED–LFD group had four consultations due to the nature of the three-phase diet: one at baseline, one at the end of the restrictive phase to assist with reintroduction, another at the end of the reintroduction phase, and a final visit 6 months after the initial consultation. The dietitians assessed each participant’s adherence to the intervention weekly through telephone calls. To evaluate the adherence of both groups to the diet, a consistent method, utilizing a 5-point Likert scale, was implemented based on the FODMAP Adherence Report Scale (FARS) [36]. Participants were asked: “How often did you follow your weekly diet during the specific evaluation period?” The response options ranged from “never, rarely, sometimes, most of the time, and every day,” corresponding to scores of 1 to 5. The maximum possible score was 25.

### 2.5. Exploratory Analysis

Biomarkers (like SCFA/BCFA) may provide insights into the biological changes in the underlying disease and may allow for objective measurements without self-reporting bias [37,38]. Additionally, diets low in FODMAP lead to a significant reduction in the fecal total SCFA in IBS patients, indicating that the low-FODMAP diet normalizes the IBS patients’ abnormal SCFA concentrations [37].

A subgroup of participants provided stool samples at each study time point to measure SCFA and BCFA levels, aiming to detect volatile compound changes during interventions that may merit further investigation as objective measures of responses to dietary interventions [27,39].

In this subgroup, dieticians closely assessed adherence to the diet, using detailed, triple-pass 24 h dietary recalls to evaluate the daily intake of macronutrients and FODMAPs for both interventions. The dietary recalls consisted of 3-day food records conducted twice, including two consecutive weekdays and a weekend day, during the two follow-ups via telephone. Trained dietitians asked participants to recall their food intake from the previous day, including all foods and beverages consumed from the moment they woke up until the following morning. The Monash FODMAP calculator was used to estimate the daily intake of FODMAPs [40].

#### SCFA and BCFA Analysis

SCFAs, the bacterial metabolites produced by fermented nondigestible carbohydrates, and BCFAs, derived from the metabolism of essential amino acids [13], were extracted and analyzed using Gas Chromatography/Mass Spectrometry (GC/MS) following the methods outlined by Zhang M et al., with minor modifications [41]. A detailed description of the methodology and statistical analysis is provided in the Supporting Information.

### 2.6. Study Outcomes

Primary outcome: change from baseline of IBS symptoms severity at the first and the second follow-up, using the IBS-SSS [42]. Co-primary endpoint: the rate of responders, defined by a ≥50-point reduction in IBS-SSS, which has been considered a clinically significant improvement [26] at the two follow-ups. Secondary endpoints included changes in symptom burden evaluated by the Gastrointestinal Symptom Rating Scale-IBS version (GSRS-IBS) [43], quality of life by the IBS-QoL questionnaire [44], and the 12-Item Short Form Survey (SF-12) [45], anxiety and depression levels measured by the Hospital Anxiety and Depression Scale (HADS) [46], and changes in stool forms measured by the BSFS. We also measured the adequate relief of IBS symptoms (AR) [47,48], adherence to dietary interventions, the use of rescue medications, the percentage of participants who achieved IBS-SSS < 175, the discontinuation rate (along with the reasons for discontinuation), as well as AEs and SAEs at both follow-up time points.

### 2.7. Statistical Analyses

The sample size calculation was based on the meta-analysis of Varju et al. [49]. Fifty-four participants per group were required to detect a significantly higher improvement in the MED–LFD group IBS-SSS (estimated increment of 100 points) compared with the NICE group (estimated increment of 59 points), with an estimated standard deviation equal to 60, and pre-specified statistical power of 80%, significance level α = 0.05, and 10% adjustment for non-adherence.

The intention-to-treat (ITT) population consisted of all randomized participants, irrespective of intervention completion. The Per-protocol (PP) population consisted of subjects who adhered to the study protocol. We performed ITT and PP analyses for IBS-SSS response and AR rate measurements. PP analysis was used for IBS-SSS, IBS-QoL, SF-12, HADS, and GSRS-IBS scores and BSFS changes, as well as the use of rescue medications. Discontinuation rate (and causes) and AE/SAEs were measured in the ITT population.

Categorical variables are presented as absolute (n) and relative (%) frequencies, and quantitative variables as mean (standard deviation) or median (interquartile range) for those not normally distributed. Normality was checked graphically (i.e., using histograms, p-p plots, boxplots, and q-q plots). The difference for each score (Δ) was calculated as the follow-up score minus the baseline score. We used paired tests within each group to assess changes in primary and secondary endpoint scores. We utilized independent Student’s *t*-test and the Mann-Whitney U test to compare the scores between the MED–LFD and NICE groups at each study time point for normally and non-normally distributed scores, respectively. We used the Chi-squared test to compare all categorical variables between the two treatment groups. Additionally, we implemented univariate and multivariate logistic regression models to detect factors predicting responders to dietary intervention. We used the statistical package STATA 16 for all analyses, and a *p*-value of ≤ 0.05 was considered statistically significant.

## 3. Results

### 3.1. Participants

**Figure 1:**
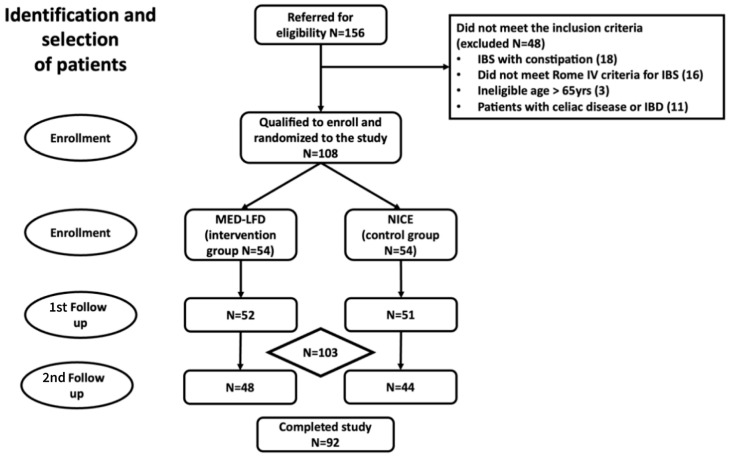
Flow chart of identification and selection of patients.

We conducted this study from June 2019 to April 2023. We screened 156 individuals, 48 of whom were not eligible (Figure 1). We enrolled patients with non-constipated IBS and randomized them into two dietary interventions. They were mostly females (77.8%) with a mean age of 40 ± 11 years. Baseline characteristics were not significantly different between the two treatment groups (Table 2). The period from baseline assessment to the first follow-up was 9.3 (range 8–11) and 4.4 (range 4–5.4) weeks for the MED–LFD and the NICE groups, respectively.

### 3.2. Overall and Individual Symptom Relief

#### 3.2.1. IBS-SSS Change in the PP Population

**Figure 2:**
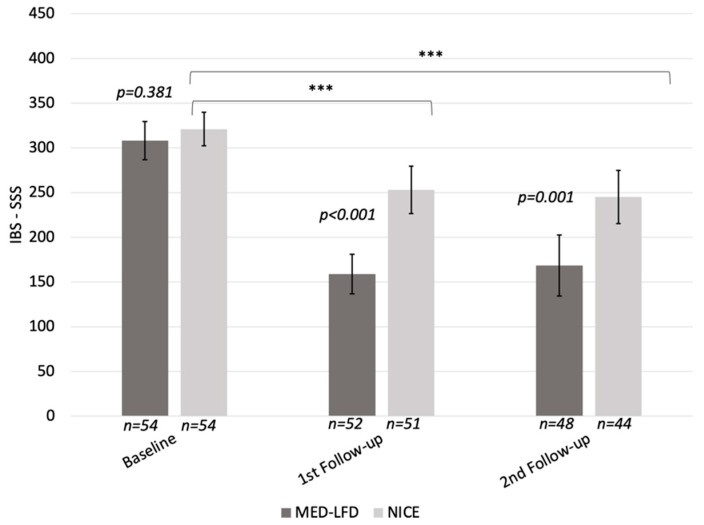
Changes in IBS-SSS between the two treatment groups. Non-significant (NS), *** *p* < 0.001 comparing the difference (follow-up—baseline) between NICE and MED–LFD groups.

We observed a significant decrease in IBS-SSS during each follow-up in both groups. The reduction in IBS-SSS was significantly greater in the MED–LFD group compared to the NICE group during the first (159 ± 80 vs. 253 ± 94; *p* < 0.001) and the second follow-ups (168 ± 117 vs. 245 ± 98; *p* = 0.001) (Figure 2).

#### 3.2.2. IBS-SSS Response Rate in the ITT and PP Populations

In the ITT analysis, the rate of MED–LFD group responders (a drop in the IBS-SSS score of more than 50 points from baseline) was significantly higher than that of the NICE group at the first [MED–LFD 44 (81.5%) vs. NICE 31 (57.4%); *p* = 0.007] and the second [MED–LFD 38 (70.4%) vs. NICE 23 (42.6%); *p* = 0.004] follow-ups. Similar intervention response rates were detected in the PP analysis: MED–LFD 44 (84.6%) vs. NICE 31 (60.8%); *p* = 0.007 and MED–LFD 38 (79.2%) vs. NICE 23 (52.3%); *p* = 0.006 at the first and second follow-ups, respectively.

#### 3.2.3. GSRS-IBS, IBS-QoL, SF-12, HADS Changes in the PP Population

As shown in Table 3, there were no significant differences in GSRS-IBS, IBS-QoL, SF-12, and HADS scores between the two groups at baseline. Both groups achieved significant improvements regarding most scores in the first (anxiety did not improve significantly in the NICE group) and second follow-ups (anxiety and depression did not significantly improve in the NICE group). At both follow-ups, MED–LFD-treated patients reported higher improvement in all total scores and most of the scores’ subscales compared with NICE group patients (Table 3).

#### 3.2.4. BSFS Changes in the PP Analysis

Patients were classified according to their BSFS type: BSFS type 1–2, type 3–5, and type 6–7 (diarrhea) (Table 4). At baseline, there was no difference between the two study groups regarding BSFS stool type; no participants reported BSFS type 1 or 2.

At the first follow-up, the percentage of patients with BSFS types 6–7 decreased from 40.74% to 9.62% in the MED–LFD group and from 52.83% to 27.45% in the NICE group. This improvement was maintained through the second follow-up. Additionally, the rate of patients in the MED–LFD group with BSFS types 3–5 was significantly higher compared to those in the NICE group (84.62% vs. 60.78% at the first follow-up and 81.25% vs. 61.36% at the second follow-up), as shown in Table 4.

#### 3.2.5. Adequate Symptom Relief in the ITT and PP Populations

The MED–LFD group reported a significantly higher AR at the first [MED–LFD 47 (87.0%) vs. NICE 35 (64.8%); *p* = 0.007] and second [MED–LFD 35 (64.8%) vs. NICE 14 (25.9%); *p* < 0.001] follow-ups, in the ITT analysis. Similarly, in the PP analysis, higher adequate symptom relief was reported in the MED–LFD compared to the NICE group at the first [MED–LFD 47 (87.0%) vs. NICE 35 (64.8%); *p* = 0.007] and second [MED–LFD 35 (72.9%) vs. NICE 14 (31.8%); *p* < 0.001] follow-ups.

### 3.3. Adherence to Intervention

Details concerning adherence to the study intervention are presented in Supplementary Figure S1. More precisely, more patients reported high adherence (most of the time or every day) in the MED–LFD group compared with NICE at both follow-ups (first: 75% for MED–LFD vs. 41% for NICE, second: 45% for MED–LFD vs. 7% for NICE; both *p*’s < 0.001). Additionally, both groups reported significantly less adherence during the second follow-up compared to the first follow-up (*p* < 0.001). Discontinuation rates were 4.6% (3.70% and 5.55% for the MED–LFD and NICE groups, respectively) at the first follow-up and 14.8% (11.1% and 18.5% for the MED–LFD and the NICE groups, respectively) at the second follow-up. During the period leading up to the first follow-up, two patients from the MED–LFD group and three patients from the NICE group discontinued the intervention due to their inability to adhere to the assigned diet. Following that, and up to the second follow-up, two patients from the MED–LFD group discontinued participation for the same reason. Additionally, three participants ceased involvement due to unspecified reasons; five were lost to follow-ups. In the NICE group, the numbers were three, five, and six for these respective categories.

### 3.4. Predictors of Dietary Intervention Response

Univariate and multivariate logistic regression models were used to assess potential predictors of intervention responders, as presented in Table 5. According to the multivariate model, the type of intervention (MED–LFD) was the strongest independent predictor of being a responder at the first (OR = 6.66; 95%CI = 1.46, 30.4) and the second (OR = 4.85; 95%CI = 1.31, 17.96) follow-ups. Other independent predictors for response at the first follow-up were baseline high IBS-SSS (OR = 1.03; 95%CI = 1.01, 1.05) and high symptoms burden score (OR = 0.12; 95%CI = 0.03, 0.46); independent predictors of response at the second follow-up were intervention adherence and baseline IBS-SSS (OR = 2.71, 95%CI = 1.57, 4.66 and OR = 1.02; 95%CI = 1.01, 1.04, respectively).

### 3.5. Safety

No SAEs were recorded during the study period, while 15 AEs were recorded (8 in the MED–LFD and 7 in the NICE group) including headache (n = 4), fatigue (n = 3), worsening of the abdominal pain (n = 2) or diarrhea (n = 5) and backache (n = 1), equally distributed in the two treatment arms. No patient discontinued study participation due to AE. Eighteen (10 and 8 in the MED–LFD and NICE groups, respectively) patients reported the use of rescue medication 26 times (12 and 14 in MED–LFD and NICE groups, respectively) during the intervention, including paracetamol (n = 10), loperamide (n = 14) and a combination of the two medications in 2 cases.

### 3.6. Exploratory Analysis Results

A random subset of 40 participants from both groups provided stool samples for the exploratory analysis. Their demographic and clinical characteristics did not differ between the two interventions, as shown in Supplementary Table S2. MED–LFD patients reported a significantly higher improvement in the IBS-SSS at the first follow-up compared to NICE patients (IBS-SSS score at the first follow-up: 184 ± 78 vs. 254 ± 120; *p* = 0.034). MED–LFD patients improved their stool form: 73.9% were classified into types 3–5 at baseline, 82.6% at the first follow-up, and 85% at the second follow-up, which was not the case for NICE patients, as shown in Supplementary Table S3.

Adherence to diet intervention did not differ significantly between the two groups during both follow-up periods. During the first follow-up, the MED–LFD group reported significantly lower lactose, sorbitol, and total oligosaccharides intake compared with the NICE group; this difference was not evident in the second follow-up (Supplementary Table S4).

#### Stool SCFAs and BCFAs Analyses

The distribution of the fecal SCFAs/BCFAs concentration levels among the two groups and time points is presented with violin plots in Supplementary Figure S2.

**Figure 3:**
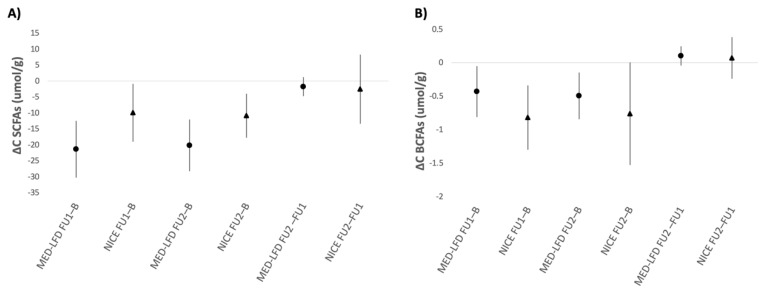
Changes from baseline levels at first and second follow-up between the two intervention groups and within each group: (**A**) total SCFAs; and (**B**) total BCFAs. Values are shown as mean (95%CI); a significant difference is detected if the 95%CI does not cross the line at 0. For example, total SCFAs stool concentration in the MED–LFD group decreases significantly (95%CI does not cross the line at 0) from baseline at the first follow-up, while there is no significant change of BCFAs stool concentration in the NICE group at the second follow-up compared to that at the first follow-up (95%CI crosses the line at 0). ΔC: change in concentrations; MED–LFD: Mediterranean Diet–Low-FODMAP Diet; NICE: National Institute for Health and Care Excellence Baseline; B: Baseline; FU1: First Follow-up; FU2: Second Follow-up.

The differences in mean concentration levels of total stool SCFAs and BCFAs from baseline between the groups are illustrated in Figure 3. In the first follow-up, we observed a significant reduction in the concentrations of total SCFAs and BCFAs in the MED–LFD (−21.4 ± 20 and −0.429 ± 0.882) and the NICE (−9.9 ± 17.7 and −0.819 ± 0.933) groups, respectively. At the second follow-up, similar reductions from the baseline concentration levels were observed in both intervention groups (total SCFAs: −20.2 ± 18.7 in MED–LFD vs. −10.9 ± 7.4 in NICE; total BCFAs: −0.494 ± 0.806 in MED–LFD vs. −0.76 ± 0.83 in NICE) (Figure 3). We observed a similar trend for each SCFA and BCFA, separately (Supplementary Figure S3).

A polynomial association was observed between the changes in IBS-SSS scores and the changes in stool concentrations of BCFAs and SCFAs from baseline to the first follow-up (Supplementary Figure S4). Specifically, there was a slight upward trend in the difference between BCFA levels, indicating that a decrease in IBS-SSS scores is associated with a reduction in BCFA levels during the first follow-up. This trend peaks around an IBS-SSS change of minus 50 points, which is the cut-off for clinical improvement. Beyond this point, the association with BCFAs slightly declines. A similar trend was noted for SCFA level changes, with the peak occurring around an IBS-SSS change of minus 100 points, after which the association diminishes.

## 4. Discussion

The study results demonstrated the superiority of MED–LFD over the NICE guidance for dietary intervention for the management of non-constipation IBS symptoms. While both diets improved patients’ symptoms, as measured by the IBS-SSS, the MED–LFD diet showed a significantly greater impact at both follow-up assessments. In detail, the observed mean reduction in IBS-SSS scores was significantly different between the MED–LFD (159 ± 80 points) and the NICE group (253 ± 94 points), yielding a net 94-point difference. This suggests that patients in the MED–LFD group experienced approximately a two-fold symptom improvement (a reduction of ≥50 points on the IBS-SSS, indicating clinically significant symptom relief) compared to the NICE group. A 100-point reduction signifies a shift from moderate to mild symptom severity, indicating that the MED–LFD intervention is more effective in helping patients to achieve a milder disease state compared to the standard NICE guidance.

One recent literature review summarized the evidence indicating that enriching the LFD with components of the MED may effectively optimize its benefits and improve the symptoms of IBS [23]. Furthermore, studies in the literature show that introducing LFD for long periods can result in insufficient intake of essential nutrients like iron, folate, magnesium, vitamins B and C, and calcium [33,50,51]. Until now, only one RCT assessed the feasibility of the Mediterranean diet across all subtypes of IBS and its beneficial impact on gastrointestinal and psychological symptoms [52]. Additionally, one non-randomized clinical study examining the clinical efficacy of the Mediterranean diet in all subtypes of IBS suggested beneficial effects for the management of gastrointestinal symptoms [53]. To the best of our knowledge, this is the first randomized controlled study aiming to assess the effectiveness of a Mediterranean version of LFD for managing the symptoms of IBS without constipation.

MED–LFD was associated with better adherence, without serious adverse events and limited use of rescue medications. Similar findings were noted in patients reporting adequate symptom relief, quality of life, symptom burden, anxiety, and depression. In our study, we identified that the type of intervention (MED–LFD diet) and the severity of IBS, as measured by IBS-SSS, were independent predictors of short-term and 6-month after-treatment intervention response. At least two recent meta-analyses agree that LFD is the dietary intervention of choice for managing IBS symptoms. However, the results are highly heterogeneous due to variability in the inclusion criteria, nutritional interventions, and primary outcomes [54,55]. Thus, we performed a literature search to identify RCTs evaluating LFD versus traditional dietary advice (including no specific recommendations, NICE recommendations, or habitual diet) with the primary endpoint being the drop of IBS-SSS at the end of the re-introduction phase of the LFD during the last decade. This search did not identify any RCT fulfilling all search criteria. However, nine RCTs involved 324 and 347 patients in the LFD and NICE diet [6,53,56,57,58,59,60,61,62], respectively, measuring IBS-SSS drop at the end of the restrictive phase only. Although we cannot directly compare our results to those of these studies, the mean benefit of our MED–LFD intervention ranks among the highest of the nine studies. Only two of the aforementioned studies evaluated the long-term effect of LFD on IBS-SSS [58,60]. Similar to our study results, but with a shorter period of observation, Goyal et al. showed that at 4 months of follow-up, the mean IBS-SSS in the LFD group was significantly lower than that of the traditional dietary advice group [58]. Harvie et al. demonstrated a noticeable reduction in IBS-SSS in the LFD group at 3 months; this reduction was maintained during the 6 months of observation [60]. While other studies have reported sustained responses to LFD for longer periods, their design and outcomes are not directly comparable to ours [63].

IBS patients often experience a lower quality of life and report a higher frequency of non-gastrointestinal symptoms in comparison to healthy individuals [64]. The use of LFD has resulted in higher QoL scores compared with traditional diets [65,66] except for “food avoidance” and “sexual” domains [67]; this finding aligns with the results of our study, and there are several speculations trying to explain this discrepancy [68]. More importantly, the IBS-QoL score showed clinically significant improvement (more than 14 points [69]) only in the MED–LFD group, at both follow-ups. Our MED–LFD diet provides enough fiber, averaging 25 g per 1800 kcal, meeting the Academy of Nutrition and Dietetics’ recommendation of 14 g of fiber per 1000 kcal. This amount of fiber is beneficial for patients with all subtypes of IBS and can contribute to overall well-being [27,70,71,72]. In addition, MED–LFD is enriched with foods containing omega-3 fatty acids, phenolic compounds, and antioxidants, whose protective role against inflammatory conditions and psychological disorders is well documented [23,27,73,74,75].

Our study highlighted significant improvements in anxiety and depression scales in the MED–LFD group; this is in line with studies showing that adherence to the MED improves depressive symptoms [76,77]. On the contrary, another study and meta-analysis revealed contradicting effects of LFD on anxiety and depression [78,79].

In our study, several mechanisms possibly contribute to reducing anxiety and depression: (1) Phenolic compounds help lower levels of IL-6 and TNF-α, which reduces neuroinflammation; (2) Restricting FODMAPs decreases visceral hypersensitivity and serotonin secretion [80], while Mediterranean foods enhance serotonin availability [81,82]; and (3) Omega-3 fatty acids and fiber help to normalize the reactivity of the HPA axis by lowering cortisol responses and improving gut permeability [83].

Some may criticize our choice not to employ triple-pass 24 h dietary recalls for accurately measuring adherence to the intervention. However, implementing this recall tool in a cohort of 100 patients can be burdensome. Therefore, we opted for a simpler tool (Likert scale), which is already established in the literature for assessing adherence to dietetic plans [36,57]. Existing studies in the literature indicate that symptomatic improvement is the major predictor of adherence to the diet [84]. Thus, it is not surprising that adherence to the MED–LFD diet surpassed that of the NICE in our study. We may also speculate that one more (three vs. two) dietetic post-baseline consultation and the longer duration of intervention in the MED–LFD group might also contribute to higher adherence [29]. Importantly, the majority of our patients receiving dietetic-led MED–LFD advice adhered to the intervention until the first follow-up; 6 months later, adherence significantly fell when they stopped receiving frequent dietetic-led advice [85].

We acknowledge that the results of our exploratory analysis are limited and should be interpreted cautiously. However, they might merit consideration for the future design of microbial volatile compounds studies in IBS [86]. While the role of BCFAs and SCFAs is complex and depends on their concentrations in the intestinal environment [12,13,87], our results showed that dietary intervention reduced SCFA/BCFA stool concentrations, suggesting a potential marker of treatment success. This aligns with previous research indicating that SCFA levels correlate with symptom severity in IBS with diarrhea [12,37,88,89].

In this randomized controlled trial, we developed an innovative dietary model that combined the benefits of LFD for reducing gastrointestinal symptoms and the anti-inflammatory properties of MED. The study used strict eligibility criteria (ROME IV, non-constipated IBS patients), specifically concerning red flag symptoms, the use of probiotics and antibiotics before the study began, and the control of rescue medications. Additionally, we ensured that our sample size was adequate based on data from one meta-analysis of LFD trials. We also studied the effects of our intervention beyond the restrictive phase of the MED–LFD to the reintroduction and personalization of symptoms study phases, while the majority of studies assessing the LFD focus on the initial short-term elimination phase [63,85]. Importantly, the novelty of our study in measuring the endpoint outcomes after completing the re-introduction phase of the MED–LFD might be considered as an additional study strength, since the lack of endpoint measurements after the FODMAPs re-introduction phase has been criticized as a methodological flaw [29]. 

Our study also has limitations. A common issue in dietary intervention trials is the lack of blinding [90], which is especially problematic when the active comparator is standard advice such as the NICE recommendations [90]. Additionally, the LFD is a well-known intervention among IBS patients; hence, this knowledge might shape the positive clinical response to a sizable extent, potentially even more than the actual intervention. Our dietary intervention was not fully controlled. No meals were given to participants, considering that controlled feeding studies do not reflect real-life eating behavior, posing questions about the same symptom response that would occur when patients tried to incorporate the intervention into their daily diet [91]. The similarity in the two groups’ access to extra virgin olive oil, the primary source of fat in Greek cuisine, which provided anti-inflammatory benefits to the entire IBS cohort, might also be a study concern. In conducting our study, we did not measure in detail the dietary intake—especially FODMAPs—at baseline and after the dietary intervention, except for the exploratory analysis subgroup; however, randomization was expected to ameliorate the effects of this omission. The length of the intervention until the first follow-up was different between the two groups; this may have introduced adherence bias and may affect the estimated causal results and overall study validity [92]. We additionally acknowledge that the synergy of MED and LFD might be confusing and challenging to learn and teach, at least at the beginning, since there is a prejudice that each of them contradicts the other, and that Mediterranean components of the MED–LFD might not be available in every household. Thus, applying both diets at the same time in real-world settings necessitates careful planning and the involvement of dietitians trained in LFD. We encountered a high rate of drop-outs during the second follow-up; however, this phenomenon is not unusual in long-term evaluations in which the percentage of patients with IBS who completed the study, out of those initially enrolled, ranged from 19% to 83% [93].

Lastly, the study was conducted at a tertiary facility, where dietitians certified by Monash University in gastrointestinal disorders guided the patients in implementing an LFD. They monitored adherence to the diet and ensured nutritional adequacy by optimizing nutrient intake and overseeing food choices, thus raising questions about the generalizability of our results in everyday practice.

## 5. Conclusions

In conclusion, our randomized controlled parallel-group design study demonstrated that our innovative dietetic intervention, a low-FODMAP diet with components of the Mediterranean diet, is superior to the NICE recommendations for the management of symptoms of IBS without constipation. It improves quality of life and reduces anxiety and depression in these patients without serious adverse events; all these effects lasted for at least 6 months. This combination offers a balanced approach to managing symptoms and promoting long-term gut health, ultimately improving QoL and overall health. The novelty of this intervention might also limit its applicability since it requires the alleviation of existing prejudices; it might also be challenging to learn and teach. Whether implementing stool volatile compounds measurement as an objective marker of intervention response remains to be clarified by future sophisticated studies.

## Figures and Tables

**Table 1:** Recommended number of servings for each food group per meal, day, and week according to the Mediterranean food pyramid, as included in the MED–LFD.

	Mediterranean Pyramid [32]	Servings	Med–LFD	Servings
Consumption in each main meal	Bread, pasta, rice, other cereals	1–2	Bread, pasta, rice, other cereals	Not in every meal (see daily consumption below)
	Fruits	1–2	Fruits	
	Vegetables	≥2	Vegetables	
Daily consumption	**Olive oil**	The major source of fat	**Olive oil**	**6**
	**Milk and Dairy products**	**2**	**Milk and Dairy products**	**3**
	**Nuts**/seeds	**1–2**	**Nuts**	**3**
	**Herbs**, **spices**	Non defined	**Herbs, spices**	**Moderate consumption**
	Garlic, onions	Non defined	**-**	**-**
	Bread, pasta, rice, other cereals	4–8	Bread, pasta, rice, other cereals	8 *
	Fruits	4–8	Fruits	4 *
	Vegetables	≥8	Vegetables	4 *
Weekly consumption	**Meat**	**2**	**Meat**	**2**
	**Eggs**	**2**	**Eggs**	**2**
	**Fish**	2	**Fish**	4
	**Poultry**	**2**	**Poultry**	**2**
	**Wine**	**Moderate consumption**	**Wine**	Moderate consumption **
	Legumes	2–3	-	-
	**Potatoes**	≤3	**Potatoes**	8
	Sweets	≤2	-	**-**

Notes. Foods marked in bold are consumed in similar frequencies and portions in both diets. The quantity and the type of fruits and vegetables consumed in the MED–LFD were determined using the FODMAP diet app [4]. * Servings were estimated for four meals daily. ** 150 mL is a serving size low in FODMAPs according to the Monash University app.

**Table 2:** Participants’ baseline characteristics per intervention group.

	Total	MED–LFD (*n* = 54)	NICE (*n* = 54)	*p*
Age (years), Mean (SD)	40 (11)	39 (11)	41 (12)	0.358
Females, *n* (%)	84 (77.8)	43 (79.6)	41 (75.9)	0.643
Educational level, *n* (%)				
Secondary	31 (28.7)	11 (20.4)	20 (37)	0.056
Tertiary	77 (71.3)	43 (79.6)	34 (63)	
Marital status, *n* (%)				
Married	47 (43.5)	18 (33.3)	29 (53.7)	0.066
Unmarried	48 (44.4)	26 (48.2)	22 (40.7)	
Divorced	11 (10.2)	8 (14.8)	3 (5.6)	
Widowed	2.1 (1.9)	2 (3.7)	0 (0)	
Income status, *n* (%)				
<500 €	37 (34.3)	18 (33.4)	19 (35.2)	
500–1000 €	40 (37)	20 (37)	20 (37)	0.971
>1000 €	31 (28.7)	16 (29.6)	15 (27.8)	
Smoking habits, *n* (%) †				
Current smoker	31 (32.0)	12 (24.5)	19 (39.6)	
Former smoker	23 (23.7)	11 (22.5)	12 (25.0)	0.174
Non-smoker	43 (44.3)	26 (53.0)	17 (35.4)	
Body Mass Index, *n* (%)				
Underweight	4 (3.7)	1 (1.9)	3 (5.6)	0.535
Normal	46 (42.6)	21 (38.9)	25 (46.3)	
Overweight	36 (33.3)	19 (35.2)	17 (31.5)	
Obese	22 (20.4)	13 (24.1)	9 (16.7)	
IBS-SSS, Mean (SD)	314 (74)	308 (79)	321 (70)	0.381

† 11 missing values; MED–LFD: Mediterranean Diet–Low-FODMAP Diet; NICE: National Institute for Health and Care Excellence; IBS-SSS: Irritable Bowel Syndrome Symptoms Severity Score.

**Table 3:** Changes from the baseline of secondary outcomes scores between the MED–LFD and NICE groups at follow-ups.

	Baseline Score			Difference from Baseline at First Follow-Up			Difference from Baseline at Second Follow-Up)		
	MED–LFD, n = 54	NICE, n = 54	*p*	MED–LFD, n = 52	NICE, n = 51	*p*	MED–LFD, n = 48	NICE, n = 44	*p*
GSRS-IBS total score, median (IQR)	4.02 (0.94)	3.95 (0.82)	0.683	−1.67 (0.99) **	−0.64 (0.95) **	<0.001	−1.53 (1.32) **	−0.5 (1.22) *	<0.001
Pain subscale	4.25 [3.50, 5.50]	4.00 [3.50, 6.00]	0.737	−2.17 (1.35) **	−0.96 (1.60) **	<0.001	−1.82 (1.64) **	−0.63 (1.76) *	0.001
Bloating subscale	5.17 [3.67, 6.00]	4.67 [4.00, 6.00]	0.763	−2.01 (1.39) **	−0.85 (1.45) **	<0.001	−1.82 (1.77) **	−0.63 (1.56) *	0.001
Constipation subscale	2.50 [1.00, 4.00]	2.00 [1.00, 3.50]	0.422	−0.48 (1.56) *	−0.16 (1.85)	0.338	−0.65 (1.66) *	−0.65 (1.76) *	0.996
Diarrhoea subscale	4.38 [3.00, 5.25]	4.50 [2.75, 5.25]	0.812	−1.97 (1.62) **	−0.82 (1.58) **	<0.001	−1.76 (1.96) **	−0.47 (1.77)	0.001
Satiety subscale	3.75 [3.00, 5.00]	3.50 [2.50, 4.50]	0.356	−1.23 (1.51) **	−0.28 (1.58)	0.003	−1.21 (1.85) **	−0.06 (1.49)	0.002
IBS-QoL total score, median (IQR)	47.5 (20.6)	49.4 (21.5)	0.649	+24.2 (20.8) **	+8.9 (12.9) **	<0.001	+26.5 (21.1) **	+11.6 (17.4) **	<0.001
Dysphoria subscale	40.6 [21.9, 60.9]	42.2 [18.8, 62.5]	0.981	+30.0 (24.8) **	+13.9 (20.0) **	<0.001	+32.4 (26.4) **	+16.9 (23) **	0.004
Interference with Activity subscale	35.7 [25, 71.4]	44.6 [28.6, 60.7]	0.629	+27.3 (25.0) **	+10.1 (15.7) **	<0.001	+29.4 (25.6) **	+10.9 (21) *	<0.001
Body Image subscale	56.3 [37.5, 68.8]	62.5 [43.8, 81.3]	0.102	+22.6 (25.8) **	+5.0 (14.5) *	<0.001	+21.4 (28.1) **	+7.4 (20.2) *	0.009
Health Worry subscale	54.2 [33.3, 66.7]	58.3 [41.7, 66.7]	0.721	+24.5 (23.4) **	+8.5 (17.8) *	<0.001	+27.8 (23.9) **	+10.1 (20.9) *	<0.001
Food Avoidance subscale	25 [8.3, 41.7]	25 [16.7, 41.7]	0.769	+10.9 (31.1) *	+5.7 (16.7) *	0.295	+14.4 (29.7) *	+4.7 (24.5)	0.093
Social Reaction subscale	56.3 [34.4, 71.9]	56.3 [43.8, 75]	0.391	+24.4 (23.7) **	+6.3 (17.5) *	<0.001	+29 (22.7) **	+13.4 (20.7) **	<0.001
Sexual subscale	50 [25, 75]	62.5 [37.5, 75]	0.769	+20.9 (30.2) **	+8.5 (20.3) **	0.017	+20.8 (27.2) **	+15.4 (29.1) *	0.361
Relationships subscale	62.5 [41.7, 79.2]	66.7 [41.7, 83.3]	0.773	+18.4 (23.8) **	+5.5 (16.8) *	0.002	+21.7 (24.5) **	+8.5 (25.1) *	0.013
HADS, mean (SD)									
Depression scale	8.10 (4.68)	8.59 (3.82)	0.565	−2.88 (4.71) **	−1.18 (3.01) *	0.035	−3 (4.29) **	−0.64 (3.61)	0.007
Anxiety scale	8.94 (4.64)	9.45 (4.67)	0.592	−2.18 (4.64) *	−0.83 (2.93)	0.092	−3.08 (4.78) **	−0.98 (4.38)	0.034
SF-12 total score, mean (SD)	40.2 (7.9)	39.3 (7.0)	0.559	+6.39 (7.97) **	+4.01 (5.78) **	0.089	+8.66 (8.23) **	+3.3 (6.61) *	0.001
Physical component	43.4 (8.6)	41.6 (9.3)	0.319	+5.54 (8.29) **	+4.73 (8.04) **	0.619	+6.65 (6.4) **	+1.34 (8.37)	0.001
Mental component	37.0 (12.0)	37.0 (9.9)	0.990	+7.24 (11.8) **	+3.29 (10.5) *	0.077	+10.67 (13.64) **	+5.26 (11.71) *	0.047

Changes in scores were defined as the difference of follow-up score minus baseline score ** *p* < 0.001; * *p* < 0.05 regarding paired tests within each group. MED–LFD: Mediterranean Diet–Low-FODMAP Diet; NICE: National Institute for Health and Care Excellence; IBS: Irritable Bowel Syndrome; HADS: Hospital Anxiety and Depression Scale; SF12: 12-Item Short Form Survey; QoL: Quality of Life; GSRS: Gastrointestinal Symptom Rating Scale; IQR: Inter-Quartile Range.

**Table 4:** Patients’ stool types, according to the Bristol Stool Form Scale, at baseline, first, and second follow-ups.

	Baseline			First Follow-Up			Second Follow-Up		
	MED–LFD	NICE	*p*	MED–LFD	NICE	*p*	MED–LFD	NICE	*p*
Patients, n	54	54	-	52	51	-	48	44	-
BSFS type, %									
1–2	0.0	0.0		5.77	11.76		8.33	4.55	
3–5	59.26	47.17	0.210	84.62	60.78	0.023	81.25	61.36	0.021
6–7	40.74	52.83		9.62	27.45		10.42	34.09	

MED–LFD: Mediterranean Diet–Low-FODMAP Diet; NICE: National Institute for Health and Care Excellence; BSFS: Bristol Stool Form Scale.

**Table 5:** Predictors of intervention response (defined by a ≥50-point reduction in IBS-SSS); univariate and multivariate analysis in the ITT population.

	First Follow-Up		Second Follow-Up	
Variables	Univariate OR (95% CI)	Multivariate OR (95% CI)	Univariate OR (95% CI)	Multivariate OR (95% CI)
Intervention (MED–LFD)	3.27 (1.36, 7.82) *	6.66 (1.46, 30.4) *	3.17 (1.44, 6.98) *	4.85 (1.31, 17.96) *
Age (years)	0.98 (0.95, 1.02)	0.96 (0.89, 1.05)	1.00 (0.96, 1.03)	1.03 (0.95, 1.11)
Gender (female)	1.89 (0.74, 4.86)	1.38 (0.28, 6.76)	0.52 (0.20, 1.36)	0.46 (0.09, 2.30)
Smoking				
Smoker	Reference	Reference	Reference	Reference
Ex-smoker	0.38 (0.12, 1.23)	0.29 (0.04, 2.04)	0.60 (0.20, 1.80)	0.44 (0.08, 2.37)
Never smoked	0.67 (0.23, 1.95)	0.38 (0.06, 2.50)	1.58 (0.62, 4.04)	0.87 (0.19, 3.96)
Education level (University)	1.12 (0.46, 2.74)	1.82 (0.33, 9.97)	1.42 (0.62, 3.28)	1.08 (0.21, 5.65)
Monthly income				
<500 €/month	Reference	Reference	Reference	Reference
500–1000 €/month	0.86 (0.32, 2.33)	3.89 (0.64, 23.67)	0.50 (0.20, 1.24)	0.54 (0.11, 2.63)
>1000 €/month	0.67 (0.24, 1.89)	2.0 (0.23, 17.13)	0.84 (0.32, 2.23)	1.48 (0.19, 11.44)
Marital status (non-married)	1.90 (0.83, 4.36)	1.14 (0.26, 5.06)	1.29 (0.60, 2.77)	1.40 (0.34, 5.80)
BMI (kg/m^2^)	1.0 (0.93, 1.06)	0.97 (0.86, 1.08)	1.03 (0.97, 1.10)	1.07 (0.97, 1.17)
Adherence to intervention	1.24 (0.89, 1.72)	1.44 (0.83, 2.50)	2.29 (1.56, 3.35) **	2.71 (1.57, 4.66) **
Baseline IBS-SSS	1.01 (1.00, 1.02) *	1.03 (1.01, 1.05) **	1.0 (0.99, 1.01)	1.02 (1.01, 1.04) *
Baseline IBS-QoL	0.97 (0.95, 0.99) *	0.96 (0.91, 1.02)	1.0 (0.98, 1.02)	0.99 (0.94, 1.03)
Baseline SF-12	0.92 (0.87, 0.98) *	1.0 (0.84, 1.2)	1.04 (0.98, 1.10)	1.1 (0.93, 1.3)
Baseline GSRS-IBS	1.04 (0.63, 1.70)	0.12 (0.03, 0.46) *	1.15 (0.73, 1.80)	0.46 (0.16, 1.31)
Baseline HADS Anxiety	1.02 (0.93, 1.13)	0.96 (0.77, 1.19)	0.94 (0.86, 1.03)	0.92 (0.77, 1.11)
Baseline HADS Depression	1.07 (0.96, 1.19)	0.91 (0.68, 1.21)	0.91 (0.82, 0.99) *	0.95 (0.74, 1.21)

** *p* < 0.001; * *p* < 0.05. A 5-point Likert scale measured adherence, with higher scores indicating better adherence. MED–LFD: Mediterranean Diet–Low-FODMAP Diet; OR: Odds Ratio; CI: Confidence Interval; BMI: Body Mass Index; IBS: Irritable Bowel Syndrome; HADS: Hospital Anxiety and Depression Scale; SF12: 12-Item Short Form Survey; QoL: Quality of Life; GSRS: Gastrointestinal Symptom Rating Scale.

## Data Availability

The data presented in this study are available from the corresponding author upon request. The data is not publicly available due to privacy/legal/ethical reasons.

## Supplementary Materials

The following supporting information can be downloaded at: https://www.mdpi.com/article/10.3390/nu17091545/s1, Figure S1: Adherence to diets; Figure S2: Acetic Acid (A), Propionic Acid (B), Butyric Acid (C), total SCFAs (D), Isobutyric Acid (E), Isovaleric Acid (F) and total BCFAs (G) levels between the subsample groups (MED-LFD, NICE) and within groups (baseline, 1st follow-up, and 2nd follow-up); Figure S3: Changes from baseline levels at 1st and 2nd follow-up between the two intervention groups and within each group of the subsample: (A) Acetic Acid, (B) Propionic Acid, (C) Butyric Acid, (D) Isobutyric Acid and (E) Isovaleric Acid. Values are shown as mean (95%CI); a significant difference is detected if 95%CI does not cross the line at 0; Figure S4: Association between BCFAs and SCFAs levels change with IBS-SSS change from baseline at the 1st follow-up in the subsample; Table S1: Weekly meal plans for the MED-LFD (A) and NICE (B) groups, respectively; Table S2: Characteristics of the two groups for the exploratory analysis; Table S3: Patients' stool type, according to the Bristol stool form scale, at baseline, 1st, and 2nd follow-ups; Table S4: The average FODMAP intake per meal per intervention group of the subsample, based on their 24-h recalls at 1st and 2nd follow-ups. References [94,95,96,97,98,99,100] are cited in Supplementary Materials.

## Abbreviations

AE	Adverse Event
BCFA	Branched Chain Fatty Acids
BSFS	Bristol Stool Form Scale
FODMAP	Fermentable oligo-, di-, monosaccharides and polyols
GC/MS	Gas Chromatography/Mass Spectrometry
GSRS-IBS	Gastrointestinal Symptom Rating Scale-IBS version
HADS	Hospital Anxiety and Depression Scale
HADS-D	The HADS questionnaire measures depression
HADS-A	The HADS questionnaire measures anxiety
IBS	Irritable Bowel Syndrome
IBS-SSS	IBS severity scoring system
ITT	Intention-to-treat population
QoL	Quality of Life
LFD	Low-FODMAP Diet
MED	Mediterranean Diet
MED–LFD	Mediterranean Diet–Low-FODMAP Diet
MCS	Mental Composite Score
NICE	National Institute for Health and Care Excellence
PCS	Physical Composite Score
PP	Per Protocol
SAE	Serious Adverse Event
SF12	12-Item Short Form Survey
SCFAs	Short-chain Fatty Acids
