# Supplementary material for: Clinical Trial: A Mediterranean Low-FODMAP Diet Alleviates Symptoms of Non-Constipation IBS—Randomized Controlled Study and Volatomics Analysis
Source: Nutrients. 2025 Apr 30;17(9):1545. doi: 10.3390/nu17091545 (PMC12073482; doi:10.3390/nu17091545)
Supplement: Supplementary file 1 [file nutrients-17-01545-s001.zip › nutrients-3608014-supplementary.pdf]

SUPPORTING INFORMATION

Exploratory Analyses

We present the characteristics of patients included in the exploratory analyses in Supplemental Tables S1 and S2. In this subgroup of study participants, detailed measurement of the consumed FODMAPs per day was assessed using a triple-pass 24-hour dietary recall (on consecutive days, including a weekend day) twice[1] at the two follow-ups by telephone contact.

Stool SCFAs and BCFAs level measurements

Fecal preparation before GC/MS analysis

In brief, aliquots (30 mg) of each fecal sample were accurately weighed into 2 mL tubes with O-ring screw caps (Sarstedt, Germany). Glass beads (1.0 mm, Sigma Aldrich, Germany) were added. The container was mixed with 300 µL HyPure water (HyClone™ HyPure Water for Injection (WFI) Quality Water, Cytiva, United States) and homogenized for 20 s under 6500 rpm with a pause of 30 s for three times using Precellys Evolution Touch homogenizer (Bertin Technologies, Montigmy le Bretonnexux, France). Then, the tubes were incubated at 4 °C with shaking for 30 min and centrifuged for 30 min at 13,000×g (4200 Tabletop Centrifuge, Kubota, Japan). 100 µL of supernatant (fecal homogenate) was transferred into a new 0.5 mL microtube pre-filled with 10 µL of 5 M HCl (Sigma Aldrich, Germany) to bring the pH of the fecal solution to 2. The acidified fecal homogenate was extracted by adding 100 µL of anhydrous diethyl ether (DE: ACS reagent, anhydrous, ≥99.0%, Sigma Aldrich, Germany), vortexed and incubated on ice for 5 min, and then centrifuged for 5 min at

10,000×g. The DE layer was transferred to a new microtube containing anhydrous sodium sulfate (Na_2SO_4; Penta Chemicals, Prague, Czech Republic) to remove the residual water. The remaining aqueous layer was further extracted with DE two more times, and the DE extracts were pooled and mixed. The process of derivatization involved transferring 100 µL of the DE extract into a 0.1 mL glass micro insert (Shimadzu, Kyoto, Japan) in a 2 mL GC vial after adding 5 µL of BSTFA (N, O-Bis(trimethylsilyl)trifluoroacetamide; Sigma Aldrich, Germany). The mixture was then kept in the GC vial capped tightly with a screw cap, vortexed for 5 s, and incubated at 37 °C for 2 h. Then, derivatized samples were loaded to GC/MS. Pure water was used for blank sample preparation to correct the background[2,3].

GC/MS Analysis

GC/MS analysis was performed by a Shimadzu QP-2010 (Shimadzu Inc., Kyoto, Japan) operated with the accompanied GC/MS Solution software. Helium was used as a carrier gas at a column flow of 1mL/min (constant linear velocity of 36.1 cm/s). Sample injections (1 µl of the derivatized sample) were performed with AOC 20i autosampler with a 3 min solvent delay time and split ratio of 10:1. The number of rinses with solvents pre- and post-run was 12, with 4 rinses per sample, plunger speed was medium, and injection pot dwell time 3 sec. Acetone, pyridine, and DE were used as solvents A, B, and C, respectively, all from Sigma, Aldrich. The temperatures of the injection port, ion source, and the GC/MS interface were 260, 230, and 280 °C, respectively. The separation of compounds was carried out in a MEGA-5HT fused silica capillary column (30 m × 0.25 mm, 0.25 µm film thickness, MEGA S.r.l., Legnano, Italy). The initial column temperature was 40 °C and held for 2 min, ramped to 150 °C at the

rate of 15 °C/min and held for 1 min, and then finally increased to 285 °C at the rate of 30 °C/min and kept at this temperature for 10 min. The ionization was carried out in the electron impact (EI) mode at 70 eV. The MS data were acquired in full scan mode from m/z 40–400. The samples were analyzed in duplicate in a predetermined order[2,3].

Data processing

The GC/MS raw data obtained from fecal samples were processed (peak identification, integration, and quantification) using the Labsolution Postrun analysis software (Shimadzu Inc., Kyoto, Japan)[4]. The identification of compounds was confirmed by injection of pure standards with the same method and comparison of the retention time and corresponding MS spectra with the NIST14/2014/EPA/NIH database[5]. The analytes were quantified in the selected ion monitoring (SIM) mode using the target ion and confirmed by confirmative ions[3]. The target ion (m/z) of acetic, propionic, isobutyric, butyric, and isovaleric acids are 117, 131, 145, 145, and 159, respectively. All standards were obtained from Sigma Aldrich, Germany.

The concentration of each organic acid (in millimolars) was determined using the external standard method. Calibration curves of analytes were constructed by preparing seven different concentration levels from a standard stock solution of the organic acid mixture. These curves were obtained by plotting the area of quantitation ions of each analyte against the nominal concentration of the calibration solution. Linear regression analysis was applied to determine the best-fit curve, with all compounds exhibiting a correlation coefficient (R^2) greater than 0.995. Further, the total SCFAs were calculated as the sum of acetate, propionate, and butyrate, while total BCFAs were defined as the sum of isobutyrate and isovalerate. All concentrations

were subsequently converted to µmol/g. The results for each sampling point were expressed as the mean.

Statistical analyses

SCFAs and BCFAs levels were measured in umol/g of stool. The final output was a data matrix with rows (fecal samples) and columns (concentration of the respective organic acids). A paired/unpaired t-test was performed to illustrate the organic acid changes among and within two groups with a statistical significance $P < 0.05$ and/or 0.01. In multiple groups comparison, significant differences between means were found using one-way analysis of variance (ANOVA), and if $P < 0.05$, Tukey's post hoc HSD test was used to determine whether the difference was significant. Differences between the treatments and baseline for each variable were graphically represented using Microsoft Excel (Office 365). Dots indicate the average of each difference and error bars the 95% confidence intervals. Hierarchical cluster analysis was performed on the data to explore the relationship between variables (organic acids) and fecal samples (dietary interventions) using Euclidean distance as the similarity measure and Ward's linkage as a clustering algorithm. The results were graphically illustrated in the form of a heatmap. MetaboAnalyst 6.0 was used for metabolomic-related statistical analysis [6].

A polynomial regression model was used to describe the relation between BCFAs/SCFAs levels change with IBS-SSS change from baseline at the 1st follow-up using polynomial analysis [7].

References

1. NIH. 24-hour Dietary Recall (24HR) At a Glance | Dietary Assessment Primer. 2024. Accessed December 24, 2024. https://dietassessmentprimer.cancer.gov/profiles/recall/

2. Hoving LR, Heijink M, van Harmelen V, van Dijk KW, Giera M. GC-MS Analysis of Short-Chain Fatty Acids in Feces, Cecum Content, and Blood Samples. *Methods Mol Biol*. 2018;1730:247-256. doi:10.1007/978-1-4939-7592-1_17

3. Clench MR, Tetler LW. CHROMATOGRAPHY: GAS | Detectors: Mass Spectrometry. In: Wilson ID, ed. *Encyclopedia of Separation Science*. Academic Press; 2000:448-455. doi:10.1016/B0-12-226770-2/00191-5

4. Shimadzu. LabSolutions Series. 2024. Accessed December 24, 2024. https://www.shimadzu.com/an/products/software-informatics/labsolutions-series/index.html

5. SIS. NIST/EPA/NIH MS/MS Mass Spectral Library 2014. 2014. Accessed December 24, 2024. https://www.sisweb.com/software/nist-msms-2014.htm

6. Pang Z, Lu Y, Zhou G, et al. MetaboAnalyst 6.0: towards a unified platform for metabolomics data processing, analysis and interpretation. *Nucleic Acids Research*. 2024;52(W1):W398-W406. doi:10.1093/nar/gkae253

7. Rawlings JO, Pantula SG, Dickey DA, eds. Polynomial Regression. In: *Applied Regression Analysis: A Research Tool*. Springer; 1998:235-268. doi:10.1007/0-387-22753-9_8

SUPPLEMENTARY Figure S1. Adherence to diets.

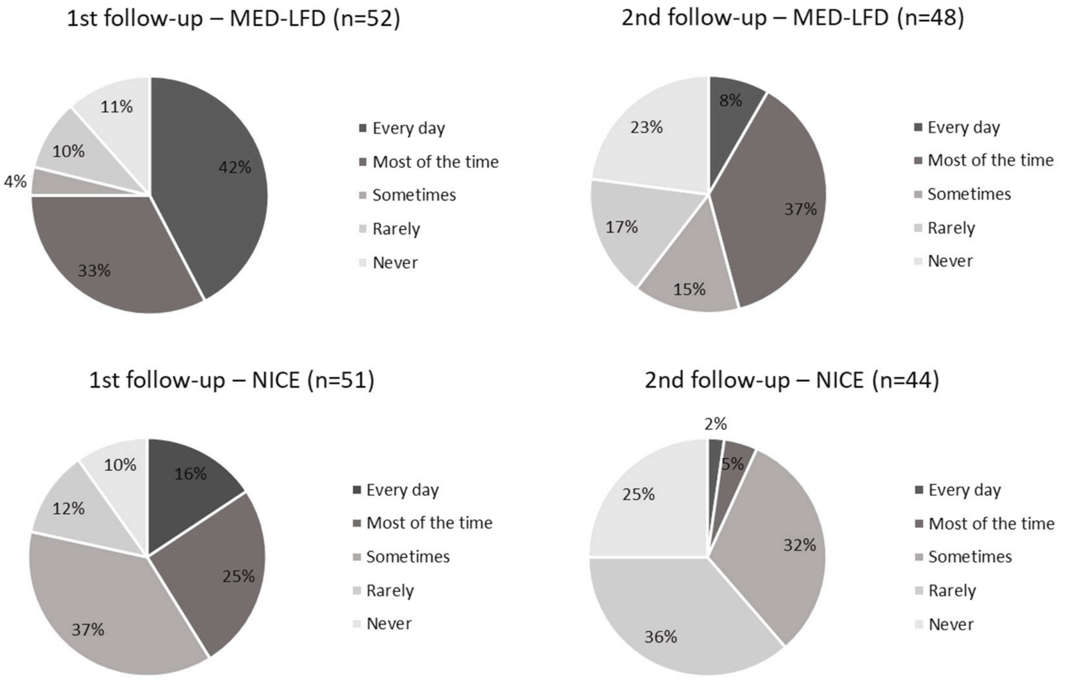

SUPPLEMENTARY Figure S2. Acetic Acid (**A**), Propionic Acid (**B**), Butyric Acid (**C**), total SCFAs (**D**), Isobutyric Acid (**E**), Isovaleric Acid (**F**) and total BCFAs (**G**) levels between the subsample groups (MED-LFD, NICE) and within groups (baseline, 1st follow-up, and 2nd follow-up).

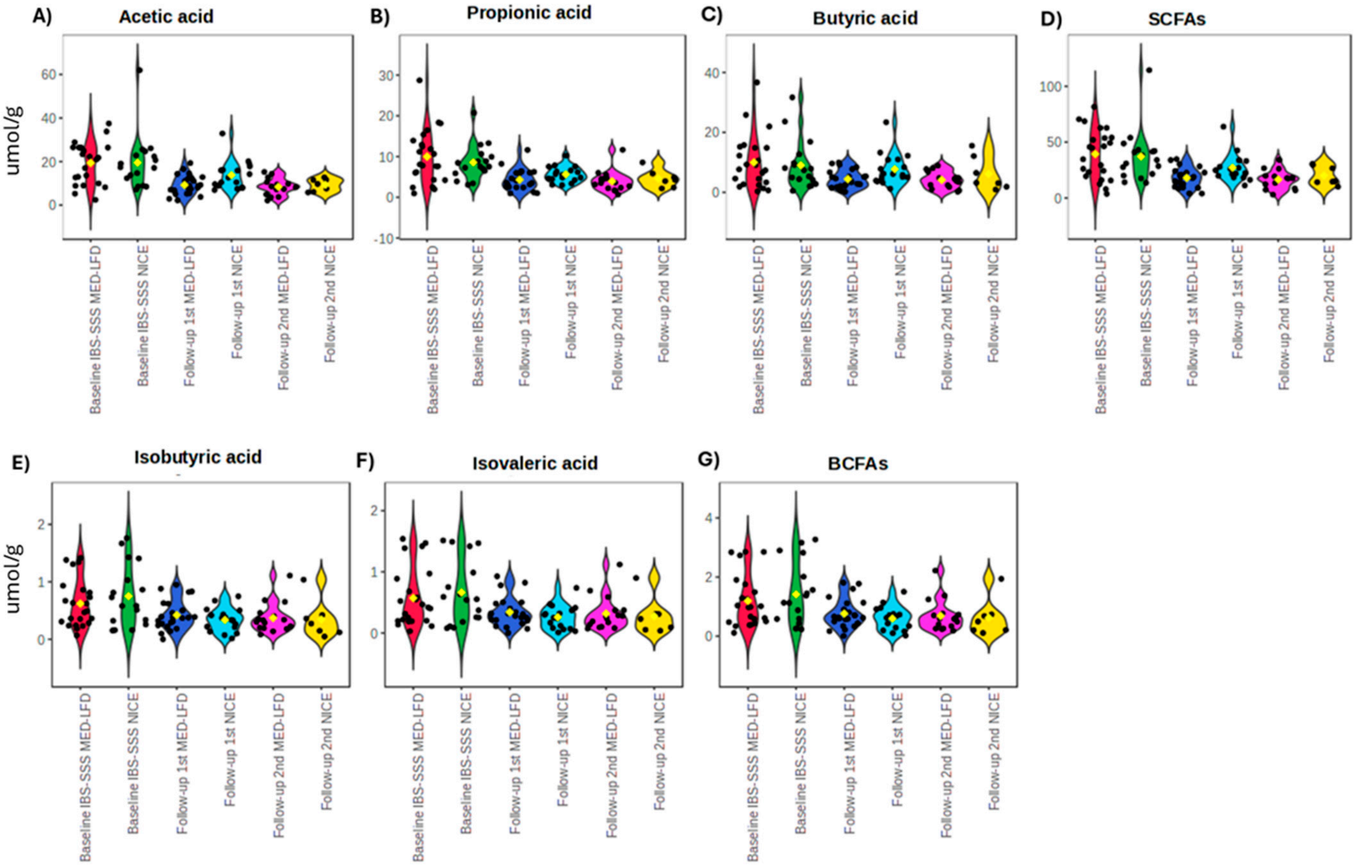

SUPPLEMENTARY Figure S3. Changes from baseline levels at 1st and 2nd follow-up between the two intervention groups and within each group of the subsample: (**A**) Acetic Acid, (**B**) Propionic Acid, (**C**) Butyric Acid, (**D**) Isobutyric Acid and (**E**) Isovaleric Acid. Values are shown as mean (95%CI); a significant difference is detected if 95%CI does not cross the line at 0.

MED-LFD: Mediterranean Diet-Low FODMAP Diet; NICE: National Institute for Health and Care Excellence Baseline; B: Baseline; FU1: 1st Follow-up; FU2: 2nd Follow-up..

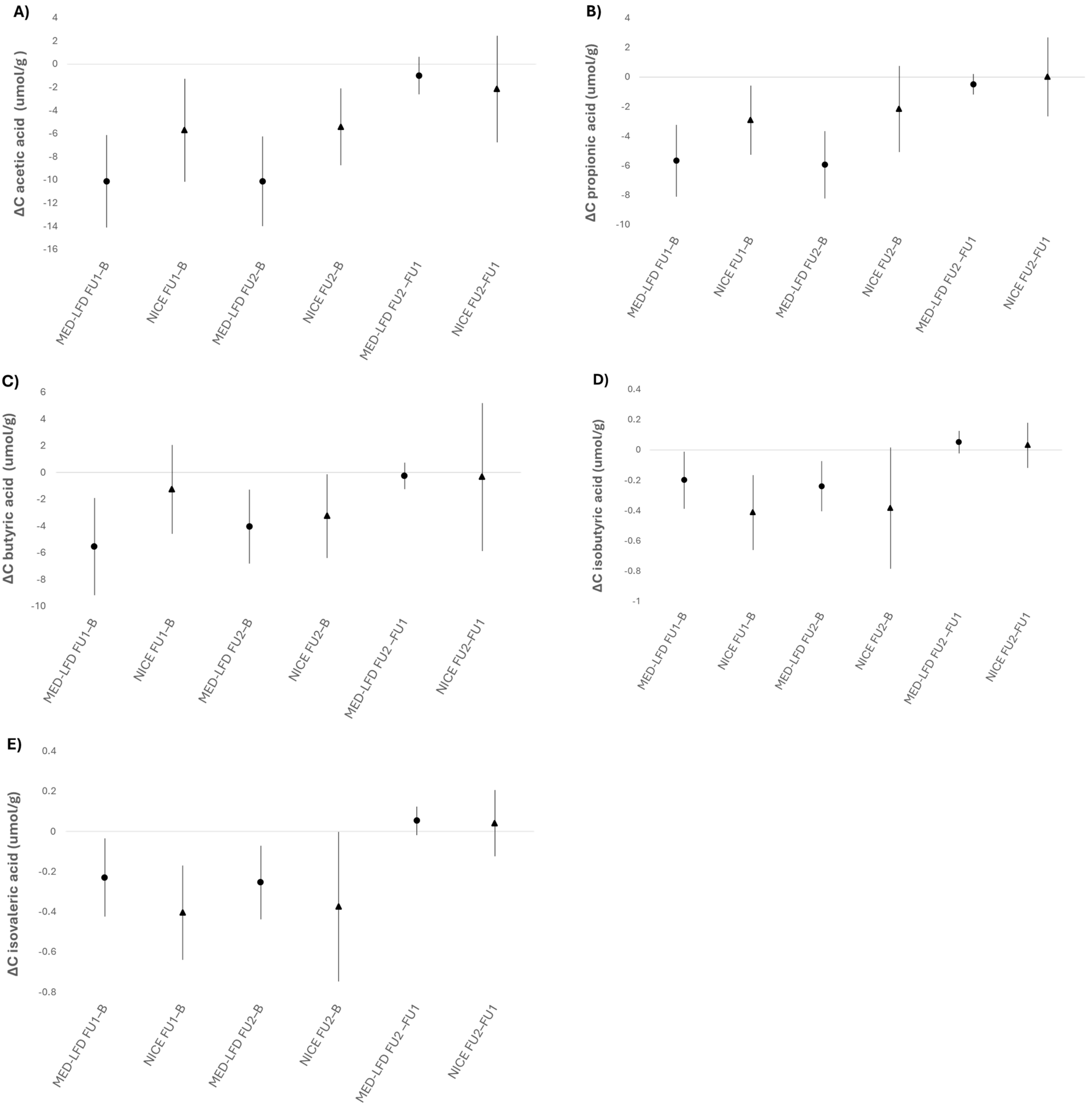

SUPPLEMENTARY Figure S4. Association between BCFAs and SCFAs levels change with IBS-SSS change from baseline at the 1st follow-up in the subsample.

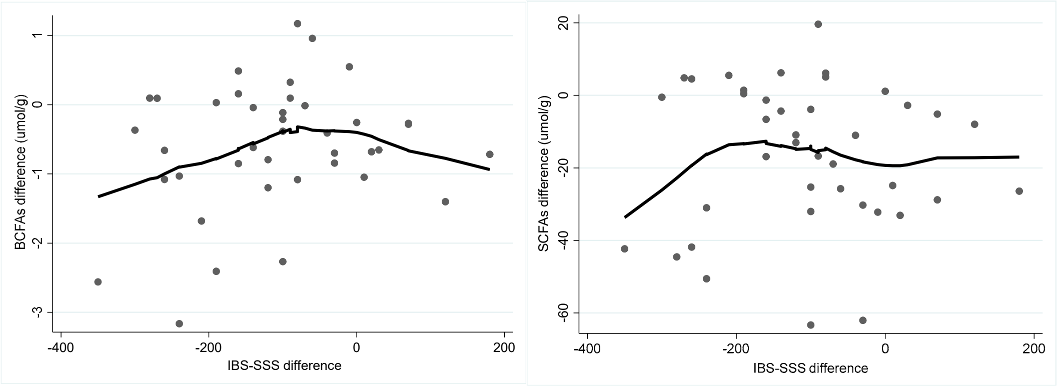

SUPPLEMENTARY Table S1. Weekly meal plans for the MED-LFD (A) and NICE (B) groups, respectively.

A. MED-LFD					
**DAY**	**BREAKFAST**	**SNACK**	**LAUNCH**	**SNACK**	**DINNER**
**DAY 1**	• 2 slices of sourdough bread (60g)<br>• 20g tahini or peanut butter		• 70g okra boiled<br>• 160g roasted potatoes<br>• 120g roasted chicken<br>• 75g cabbage + 75g carrot salad<br>• 3 tsp olive oil		• 70g okra boiled<br>• 160g roasted potatoes<br>• 120g roasted chicken<br>• 75g cabbage + 75g carrot salad<br>• 3 tsp olive oil
**DAY 2**	• 1 cup of 1.5% lactose-free milk or non-dairy alternative to milk<br>• 42g cornflakes	1 kiwi (150g) + 12 almonds (15g)	• 120g grilled fish<br>• 200g fried potatoes<br>• Greek salad (65g tomato, 75g cucumber, 75g green pepper)<br>• 3 tsp olive oil for the fish and salad	1 unripe banana (112g) or 1 orange (130g)	• 120g grilled fish<br>• 200g fried potatoes<br>• Greek salad (65g tomato, 75g cucumber, 75g green pepper)<br>• 3 tsp olive oil for the fish and salad
**DAY 3**	• 3 rice cakes (42g)<br>• 20g peanut butter<br>• 20g strawberry jam		• 2 stuffed peppers with rice (200g)<br>• 90g low-fat cheese<br>• 75g cabbage + 75g carrot salad<br>• 3 tsp olive oil<br>• 1 boiled egg		• 2 stuffed peppers with rice (200g)<br>• 90g low-fat cheese<br>• 75g cabbage + 75g carrot salad<br>• 3 tsp olive oil
**DAY 4**	• 3 rice cakes (42g)<br>• 20g peanut butter<br>• 20g strawberry jam		• 120g roasted beef<br>• 200g rice pilaf<br>• 75g green beans, boiled<br>• Greek salad (65g tomato, 75g cucumber, 75g green pepper)<br>• 3 tsp olive oil		• 120g roasted beef<br>• 200g rice pilaf<br>• 75g green beans, boiled<br>• Greek salad (65g tomato, 75g cucumber, 75g green pepper)<br>• 3 tsp olive oil
**DAY 5**	• 200ml 1.5% lactose-free milk or non-dairy alternative to milk<br>• 42g cornflakes		• 75g fried eggplants<br>• 160g roasted potatoes<br>• 100g grilled fish<br>• Greek salad (65g tomato, 75g cucumber, 75g green pepper)<br>• 3 tsp olive oil for the fish, potatoes, and salad		• 75g fried eggplants<br>• 160g roasted potatoes<br>• 100g grilled fish<br>• Greek salad (65g tomato, 75g cucumber, 75g green pepper)<br>• •3 tsp olive oil for the fish, potatoes, and salad

**DAY 6**	• 3 rice cakes (42g)<br>• 20g peanut butter<br>• 20g strawberry jam		• 150g grilled chicken<br>• 180g rice pilaf<br>• 75g cabbage + 75g carrot salad<br>• 3 tsp olive oil		• 150g grilled chicken<br>• 180g rice pilaf<br>• 75g cabbage + 75g carrot salad<br>• 3 tsp olive oil
**DAY 7**	• 1 cup of 1.5% lactose-free milk or non-dairy alternative to milk<br>• 42g cornflakes		• 240g roasted potatoes<br>• 90g low-fat cheese<br>• Greek salad (65g tomato, 75g cucumber, 75g green pepper)<br>• 3 tsp olive oil		• 240g roasted potatoes<br>• 90g low-fat cheese<br>• Greek salad (65g tomato, 75g cucumber, 75g green pepper)<br>• 3 tsp olive oil

Clarifications & Instructions

We advised patients against consuming onions and garlic. At the same time, we strongly recommended consuming oregano, thyme, cumin, and rosemary. Moderate consumption of wine was allowed (2-3 glasses per week), up to 149g.

We advised patients to consume fish 2 to 3 times per week and to limit red meat to once per week. The dietary plan provided examples of food choices and portion sizes, which were adjusted to each patient's energy needs and personal preferences.

B. NICE					
**DAY**	**BREAKFAST**	**SNACK**	**LAUNCH**	**SNACK**	**DINNER**
**DAY 1**	• 1 cup of Milk, 1% fat<br>• Breakfast cereal, Oat and Honey	1 Orange	• 180g Risotto, white rice & vegetables<br>• 100g Turkey, roasted<br>• 250g Green Salad<br>• 30g Bread, white, sliced<br>• 15g Cheese, reduced fat<br>• 3 tsp oil	1 Apple	• 180g Risotto, white rice & vegetables<br>• 120g Turkey, roasted<br>• 250g Green Salad<br>• 30g Bread, white, sliced<br>• 15g Cheese, reduced fat<br>• 3 tsp oil
**DAY 2**	• 1 cup of Milk, 1% fat<br>• with Porridge	1 banan1 Banana	• 120g grilled fish<br>• 200g fried potatoes<br>• 250g green salad<br>• 30g Bread, white, sliced<br>• 3 tsp oil	Yogurt, reduced fat, and Honey	• 120g grilled fish<br>• 200g fried potatoes<br>• 250g green salad<br>• 30g Bread, white, sliced<br>• 3 tsp oil
**DAY 3**	• 1 cup of Milk, 1% fat<br>• Breakfast cereal, cornflakes	Orange juice	• 200g Okra with tomatoes and onion<br>• 60g Bread, white, sliced<br>• 200g green salad<br>• 3 tsp oil	Yogurt, reduced fat, and 1 apple<br><br>Apples	• 150g. Chicken roasted<br>• 200g. Pasta, white, spaghetti<br>• 200g green salad<br>• 3 tsp oil
**DAY 4**	• 1 cup of Milk, 1% fat,<br>• Breakfast cereal, cornflakes	1 1 Banana	• 250g Soup, lentils<br>• 80g Cheese, Feta<br>• 60g Bread, white, sliced<br>• 3 tsp oil	1 Pear	• 250g. Spaghetti in tomato sauce<br>• 30g. Bread, white, sliced<br>• 300g. Salad, green<br>• 3 tsp oil
**DAY 5**	• 1 cup of Milk, 1% fat,<br>• Breakfast cereal, cornflakes	Yogurt, reduced fat<br>1 Apple	• 150g. Beef, fillet steak<br>• 250g Pilaf, rice with tomato<br>• 250g Salad with cabbage<br>• 30g Bread, white, sliced<br>• 3 tsp oil	Apple juice	• 200g Spaghetti Bolognese (with beef)<br>• 200g Salad with cabbage<br>• 3 tsp oil
**DAY 6**	• 1 cup of Milk, 1% fat<br>• Breakfast cereal, honey coated	1 Pear	• 250g baked beans, canned in tomato sauce<br>• Egg whole, fried<br>• 250g Salad, tomato and onion<br>• 30g Bread, white, sliced<br>• 3 tsp oil	Yogurt, reduced fat, and 80 gr. Strawberries	• 90g Chicken casseroled,<br>• 250g Potatoes,<br>• 250g Broccoli, green, boiled<br>• 30g Bread, white, sliced<br>• 3 olive oil

**DAY 7**	• 1 cup of Milk, 1% fat<br>• Breakfast cereal, cornflakes	30 gr. Biscuits, cheese-flavored, 1 Banana	• 120g Fish, grilled<br>• 300g Casserole vegetables<br>• 60g Bread, white, sliced<br>• 3 tsp oil	Yogurt, reduced fat, and 1 Pear	• 250g Spaghetti in tomato sauce<br>• 30g Cheese, Feta<br>• 300g green salad<br>• 60g Bread, white, sliced<br>• 3 tsp oil

In the NICE group, patients could freely choose food from specific categories, such as vegetables, fruits, and grains. The dietary plan provided examples of food choices and portion sizes, which were adjusted to each patient's energy needs and personal preferences.

Patients were advised to avoid missing meals or leaving long gaps between eating, and reducing intake of artificial sweeteners, and processed or re-cooked foods.

SUPPLEMENTARY Table S2. Characteristics of the two groups for the exploratory analysis.

		MED-LFD	NICE	*p*
Patients, n		23	17	-
Age, Mean (SD)		40 (12)	41 (13)	0.858
Females, n (%)		16 (70)	13 (77)	0.629
Educational level, *n (%)*				
	Secondary (high school)	3 (13)	6 (35)	*0.096*
	Tertiary (university)	20 (87)	11 (65)	
Marital status, *n (%)*				
	Married	8 (35)	9 (53)	*0.467*
	Unmarried	10 (44)	7 (41)	
	Divorced	4 (17)	1 (6)	
	Widowed	1 (4)	0 (0)	
Income status, *n (%)*				
	<500€	7 (30.5)	6 (35)	*0.882*
	500-1,000€	9 (39)	7 (41)	
	>1,000€	7 (30.5)	4 (24)	
Smoking habits, *n (%)*				
	Current smoker	5 (22)	6 (35)	*0.569*
	Former smoker	5 (22)	4 (24)	
	Non-smoker	13 (56)	7 (41)	
Body Mass Index, *n (%)*				
	Underweight	0 (0)	2 (12)	*0.259*
	Normal	12 (52)	6 (35)	
	Overweight	8 (35)	5 (29)	
	Obese	3 (13)	4 (24)	
IBS-SSS baseline, *Mean (SD)*		313 (85)	331 (72)	*0.494*
IBS-SSS 1st follow-up, *Mean (SD)*		184 (78)	254 (120)	*0.034*
IBS-SSS 2nd follow-up, *Mean (SD)*		192 (96)	241 (100)	*0.156*

Abbreviations. MED-LFD: Mediterranean version of the low FODMAP Diet; NICE: National Institute for Health and Care Excellence; IBS-SSS: Irritable Bowel Syndrome Symptoms Severity Score

SUPPLEMENTARY Table S3. Patients' stool type, according to the Bristol stool form scale, at baseline, 1st, and 2nd follow-ups

	Baseline				1st Follow-up				2nd Follow-up			
	Overall	MED-LFD	NICE	*p*	Overall	MED-LFD	NICE	*p*	Overall	MED-LFD	NICE	*p*
***BSFS type*, %**												
1-2	0	0	0		12.5	8.7	17.7		8.6	5	13.3	
3-5	70	73.9	64.7	*0.530*	75	82.6	64.7	*0.434*	71.4	85	53.3	*0.121*
6-7	30	26.1	35.3		12.5	8.7	17.7		20	10	33.3	

MED-LFD: Mediterranean version of the low FODMAP Diet; NICE: National Institute for Health and Care Excellence; BSFS: Bristol Stool Form Scale.

SUPPLEMENTARY Table S4. The average FODMAP intake per meal per intervention group of the subsample, based on their 24-hour recalls at 1st and 2nd follow-ups.

	**1st Follow-up**			**2nd Follow-up**		
**FODMAPs, *M(IQR)***	**MED-LFD (N=20)**	**NICE (N=17)**	***p***	**MED-LFD (N=19)**	**NICE (N=15)**	***p***
Excess fructose, g/meal	0.17 (0.13-0.38)	0.19 (0.11-0.83)	0.563	0.22 (0.11, 0.39)	0.3 (0.2, 0.47)	0.275
Sorbitol, g/meal	0.02 (0.0-0.06)	0.11 (0.01-0.39)	0.021	0.02 (0, 0.08)	0.14 (0.01, 0.28)	0.245
Mannitol, g/meal	0.004 (0.0-0.07)	0.06 (0.01-0.13)	0.077	0.02 (0.01, 0.13)	0.04 (0, 0.09)	0.664
Fructans, g/meal	0.43 (0.33-0.54)	0.63 (0.48-0.7)	0.051	0.54 (0.27, 0.8)	0.73 (0.51, 1.07)	0.080
GOS, g/meal	0.09 (0.07-0.18)	0.21 (0.09-0.36)	0.106	0.14 (0.07, 0.32)	0.23 (0.14, 0.48)	0.171
Total oligosaccharides, g/meal	0.54 (0.39-0.79)	0.85 (0.65-1.1)	0.038	0.7 (0.33, 1.16)	1.08 (0.72, 1.52)	0.050
Total FODMAPs, g/meal	1.36 (1.0- 1.90)	2.51 (1.68-3.42)	0.011	1.54 (1.10-2.62)	2.62 (1.42-3.90)	0.086
Lactose, g/meal	0.04 (0.01-0.56)	1.36 (0.47-2.46)	0.002	0.32 (0.04, 1.15)	0.27 (0.01, 2.39)	0.808
**Adherence, *n (%)***						
Never/Rarely	6 (26)	3 (17.5)		7 (35)	8 (53.3)	
Sometimes	0 (0)	3 (17.5)	0.173	4 (20)	5 (33.3)	0.127
Most of the time/Every day	17 (74)	11 (65)		9 (45)	2 (13.3)	

Total FODMAPs were calculated as the sum of excess fructose, sorbitol, mannitol, fructans, and total oligosaccharides. Abbreviations. M: Median; IQR: Interquartile range, g: grams; GOS: Galactooligosaccharides
